# Implementation of the FAST emergency vehicle pre-emption system may improve the outcomes of out-of-hospital cardiac arrests: a 7-year observational study

**DOI:** 10.1186/cc9712

**Published:** 2011-03-11

**Authors:** H Inaba, Y Tanaka, K Fukushima, S Tamasaku

**Affiliations:** 1 Kanazawa University Graduate School of Medicine, Kanazawa, Japan; 2 Kanazawa University Hospital, Kanazawa, Japan; 3 Kanazawa City Fire Department, Kanazawa, Japan

## Introduction

The interval of call to arrival is one of the major factors associated with good outcomes of out-of-hospital cardiac arrests (OHCAs). The FAST system helps emergency vehicles reach a scene quickly by controlling the traffic signals. The aim of study is to investigate whether the FAST system may improve the outcomes of OHCAs by decreasing the response time.

## Methods

We analyzed the data from OHCAs that were witnessed or recognized by citizens from April 2003 to March 2010. The OHCA data were compared between the two groups transported by ambulances with and without FAST units. The comparisons were made in the central and peripheral areas with and without FAST-controlled signals.

## Results

**Figure 1:**
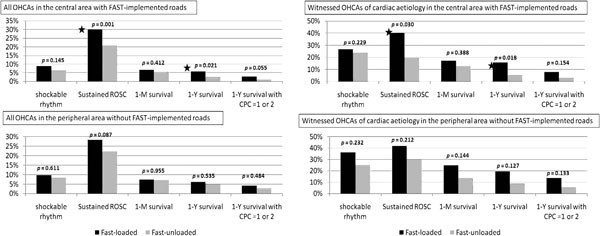
**Effect of the FAST implementation on outcomes of OHCAs in the two regions**.

Dispatch of and transportation by FAST-loaded ambulances significantly decreased the interval of call to arrival and significantly augmented the incidence of sustained ROSC and 1-year survival only in the central area (Figure 1). Monovariate analysis followed by logistic regression analysis revealed that FAST implementation is an independent factor associated with 1-year survival (adjusted odds ratio with 95% CI = 1.306 (1.014 to 1.691)) and sustained ROSC (1.249 (1.108 to 1.410)).

## Conclusions

The implementation of FAST may improve the outcomes of OHCAs mainly by reducing the interval of call to arrival.
